# Supplementary material for: Honokiol Inhibits Colorectal Cancer Cell Growth: Involvement of Hsp27 as a Molecular Target
Source: Curr Issues Mol Biol. 2025 Nov 5;47(11):921. doi: 10.3390/cimb47110921 (PMC12650836; doi:10.3390/cimb47110921)
Supplement: Supplementary file 1 [file cimb-47-00921-s001.zip › cimb-3912539-supplementary.pdf]

Supplementary Data

Results

1. Isolation of HK from Magnolia officinalis Extracts using MPLC

1.1. Solvent Fractionation and First Step Purification of HK

A total of 150 g of *Magnolia officinalis* 60% EtOH Extract was dissolved in distilled water and subjected to solvent fractionation with ethyl acetate (EA) at a 1:1 (v/v) ratio. The EA layer was collected, concentrated under reduced pressure, and freeze-dried, resulting in 3.17 g of EA fraction. The fractionation yield was calculated to be 2.11% based on the Magnolia officinalis 60% EtOH Extract (150 g). For the First step purification of HK, the recovered EA fraction (3.17 g) was dissolved in 10.5 mL of methanol to obtain a solution at an approximate concentration of 0.3 g/mL. This solution was subjected to MPLC using a silica gel column (Figure S1), resulting in three fractions, designated as Fr.1, Fr.2, and Fr.3. Among these, Fr.2 was obtained from the major peak region. The recovered amounts and purification yield for each fraction were as follows: Fr.1 yielded 19.1 mg (0.60%), Fr.2 yielded 1,330 mg (41.93%), and Fr.3 yielded 53.6 mg (1.69%).

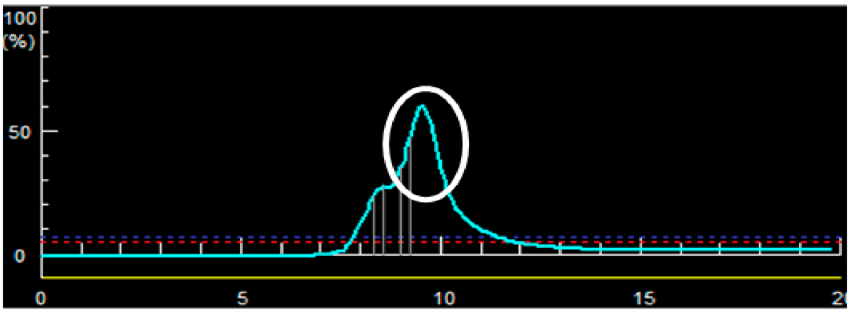

Figure S1. MPLC chromatogram of primary purification for HK. EA fraction was dissolved in methanol at a concentration of approximately 0.3 g/mL, Stationary phase was Silica gel column and Mobile phase was chloroform/methanol (94:6, v/v).

1.2. Second Step Purification of HK

Fr.2, obtained from the first step purification, was dissolved in 6.65 mL of methanol to achieve a concentration of approximately 0.2 g/mL. This solution was then subjected to secondary purification using MPLC with an ODS column (Figure S2), through which a distinct single peak was observed.

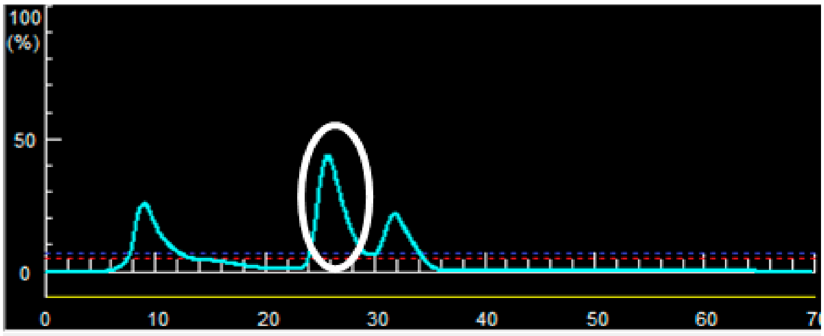

Figure S2. MPLC chromatogram of secondary purification for HK. EA fraction was dissolved in methanol at a concentration of approximately 0.3 g/mL, Stationary phase was Silica gel column and Mobile phase was chloroform/methanol (94:6, v/v)

2. *Qualitative Analysis of the Isolated Compound* (*TLC*)

Thin-Layer Chromatography (TLC) was performed to qualitatively analysis both the Magnolia officinalis 60% EtOH extract and the isolated compound (ODS Fr.1). The analysis revealed a compound with the same Rf value present in both samples. Furthermore, the isolated compound showed a single, distinct spot, indicating that it was a purified single component (Figure S3).

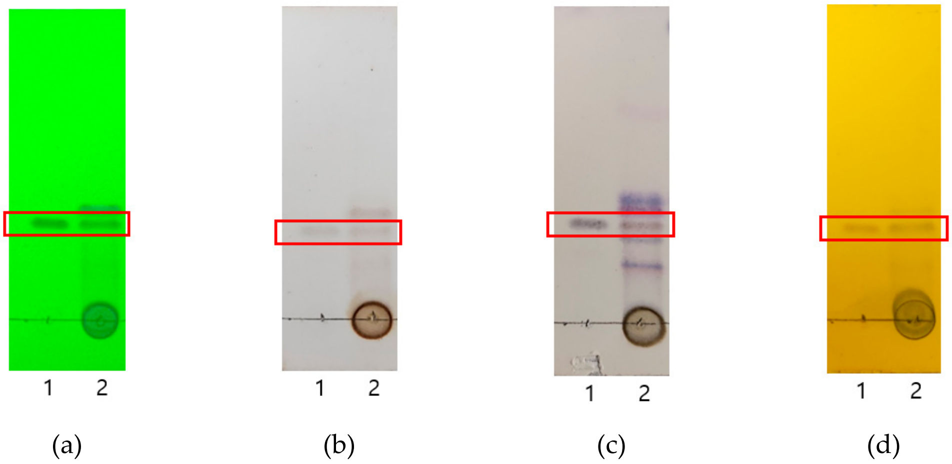

Figure S3. Thin-layer chromatography (TLC) analysis of the isolated compound (ODS Fr.1) and *M. officinalis* 60% EtOH extract. (**a**) Visualization under UV light at 254 nm; (**b**) 10% H_2SO_4; (**c**) p-anisaldehyde-H_2SO_4; (**d**) $FeCl_3$. The mobile phase was benzene/methanol (9:1, v/v). Lane 1: isolated compound (ODS Fr.1); Lane 2: *Magnolia officinalis* 60% EtOH extract. The red box indicates the region where the compound with the same Rf value was detected in both samples.

3. *HPLC Chromatographic Data by Concentration of HK*

3.1. Calibration Curve for HK Standard

Using the standard compound, peak areas were measured at six different concentrations, and a calibration curve was constructed as shown in Figure S3. The calibration equation obtained by the least-squares method was Y = 23,498X – 10,169, with a coefficient of determination (R^2) of 0.9998.

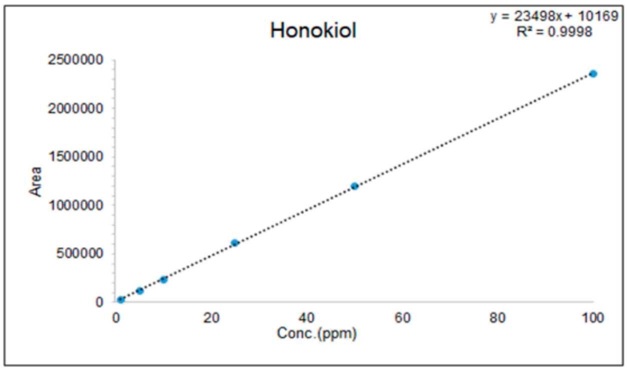

Figure S4. Calibration curve and equation for HK standard.

3.2. HPLC Chromatogram of HK Standard Data

Peaks were observed at six different concentrations, and the retention time (RT) values for the data were found to be 21.14 ± 0.03 minutes (Figure S5).

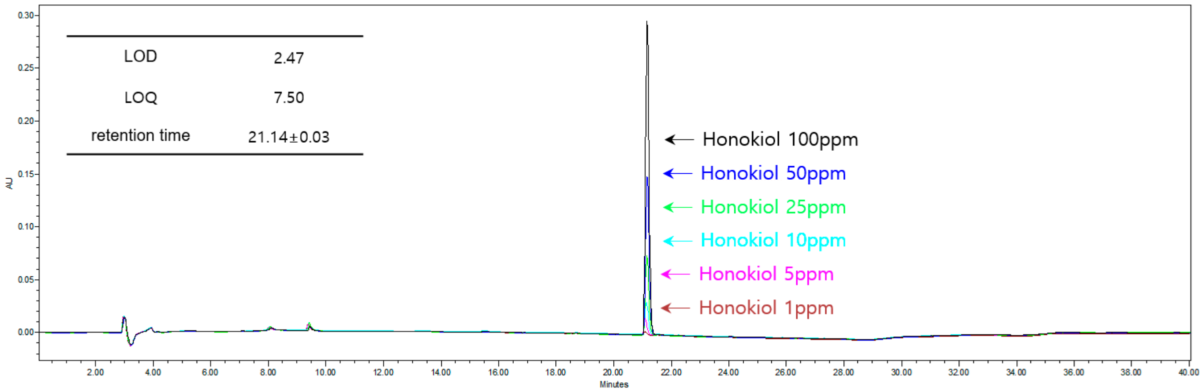

Figure S5. HPLC chromatogram of HK isolation.

4. Overexpression of Hsp27 in Various CRC cells

We confirmed that Hsp27 is over-expressed in various human CRC cell lines, all of which exhibited higher Hsp27 expression compared to CCD-18Co normal colon cells (Figure S6).

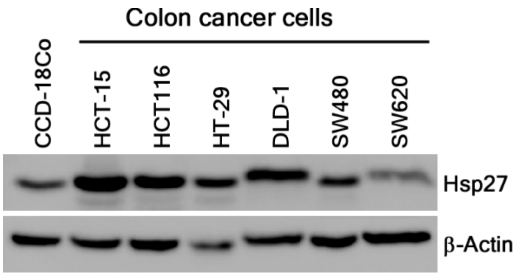

Figure S6. Expression level of Hsp27 in various CRC cells.
